# Copper and Zinc Metal–Organic Frameworks with Bipyrazole Linkers Display Strong Antibacterial Activity against Both Gram+ and Gram− Bacterial Strains

**DOI:** 10.3390/molecules28166160

**Published:** 2023-08-21

**Authors:** Sonila Xhafa, Laura Olivieri, Corrado Di Nicola, Riccardo Pettinari, Claudio Pettinari, Alessia Tombesi, Fabio Marchetti

**Affiliations:** 1 ChIP Research Center, School of Science and Technology, University of Camerino, Via Madonna delle Carceri, 62032 Camerino, MC, Italy; sonila.xhafa@unicam.it (S.X.); laura.olivieri@unicam.it (L.O.); corrado.dinicola@unicam.it (C.D.N.); 2 ChIP Research Center, School of Pharmacy, University of Camerino, Via Madonna delle Carceri, 62032 Camerino, MC, Italyclaudio.pettinari@unicam.it (C.P.); alessia.tombesi@unicam.it (A.T.)

**Keywords:** Cu(II) and Zn(II) MOFs, bis(pyrazolate) ligands, antibacterial activity, chelation theory, ROS generation

## Abstract

Here, we report a new synthetic protocol based on microwave-assisted synthesis (MAS) for the preparation of higher yields of zinc and copper in MOFs based on different bis(pyrazolyl)-tagged ligands ([M(BPZ)]_n_ where M = Zn(II), Cu(II), H_2_BPZ = 4,4′-bipyrazole, [M(BPZ-NH_2_)]_n_ where M = Zn(II), Cu(II); H_2_BPZ-NH_2_ = 3-amino-4,4′-bipyrazole, and [M_x_(Me_4_BPZPh)] where M = Zn(II), x = 1; Cu(II), x = 2; H_2_Me_4_BPZPh = bis-4′-(3′,5′-dimethyl)-pyrazolylbenzene) and, for the first time, a detailed study of their antibacterial activity, tested against Gram-negative (*E. coli*) and Gram-positive (*S. aureus*) bacteria, as representative agents of infections. The results show that all MOFs exert a broad-spectrum activity and strong efficiency in bacterial growth inhibition, with a mechanism of action based on the surface contact of MOF particles with bacterial cells through the so-called “chelation effect” and reactive oxygen species (ROS) generation, without a significant release of Zn(II) and Cu(II) ions. In addition, morphological changes were elucidated by using a scanning electron microscope (SEM) and bacterial cell damage was further confirmed by a confocal laser scanning microscopy (CLSM) test.

## 1. Introduction

The microbial infections constitute one of the most important global public health challenges of the 21st century. The misuse and abuse of antibiotics in medicine, food production and the environment as a whole have sadly further amplified this problem [1,2]. Many leading international scientific, political and social organizations have warned that the rise of antimicrobial resistance could cost the lives of millions of people if it is not tackled on a global scale [3,4]. There is a pressing need to develop new and effective antimicrobial agents that can overcome the intrinsic resistance developed against currently used biocides.

In this respect, compounds containing metal ions are promising substitutes [5,6,7,8] and, among them, porous coordination polymers, also known as metal–organic frameworks (MOFs) are attracting growing interest [9,10,11,12,13,14]. MOFs possess favourable structures of large-surface crystalline networks with regular porosity and good structural rigidity, and since their first development, they have been largely investigated in many applications in gas storage, separation of gases, purification of water, sensors, heterogeneous catalysis and drug storage and release [15,16,17,18]. Investigation of MOFs as antimicrobial agents is more recent, and the attention has been mainly paid to MOFs with Ag(I), Cu(II), Zn(II), Co(II) and Fe(III) ions. One reason for the interest in MOFs for antimicrobial applications lies in their poor or very low solubility in water and organic solvents, which allows their use in solid composite materials for a wide variety of applications [19,20,21,22,23,24]. Furthermore, the advantage of MOFs over other antimicrobial agents is that they can use different mechanisms of action, from simple surface contact with microbes through the so-called “chelation effect”, the slow and controlled release of metal ions with antimicrobial activity and/or bioactive linkers, or also by the photocatalytic production of reactive organic species (ROS) that kill microbes, and finally, thanks to their porosity, they can also act as carriers for the controlled release of biocides [25]. However, most studies on antimicrobial MOFs are related to a limited number of well-known MOFs, such as metal carboxylates and imidazolates. Moreover, despite the large number of MOFs already synthesized with essential elements Cu and Zn and used for other applications, those investigated for their potential antimicrobial activities are still relatively few [9].

We have previously synthesized and characterized several Zn(II) and Cu(II) MOFs with bis(pyrazole)-based ligands, more in detail with 4,4′-bipyrazole proligand (H_2_BPZ) [26], with bis-4′-(3′,5′-dimethyl)-pyrazolylbenzene proligand (H_2_Me_4_BPZPh), which MOFs have been investigated as potential catalysts in the peroxidative oxidation of alcohols and cyclohexane [27], and also with 3-amino-4,4′-bipyrazole proligand (H_2_BPZ-NH_2_), which MOFs have been tested as efficient carbon dioxide adsorbents [28,29]. MOFs based on H_2_BPZ were prepared through a room temperature synthesis by stirring MeOH solutions, while all the others were obtained by solvothermal methods in DMF or DMF/MeOH.

Here, we report a new synthetic protocol for MOFs of H_2_Me_4_BPZPh and H_2_BPZ-NH_2_ linkers based on the use of the MW condition to replace the previous solvothermal procedure. We considered it important to identify a methodology that requires less time, with lower energy costs and products with higher yields than the traditional solvothermal synthesis used previously. Furthermore, a detailed study of their antimicrobial activity against typical Gram-positive (*S. aureus*) and Gram-negative (*E. coli*) bacterial strains is reported for the first time on this family of MOFs. An in-depth study of their possible mechanism of action is also investigated and discussed.

## 2. Results and Discussion

**Scheme 1:**
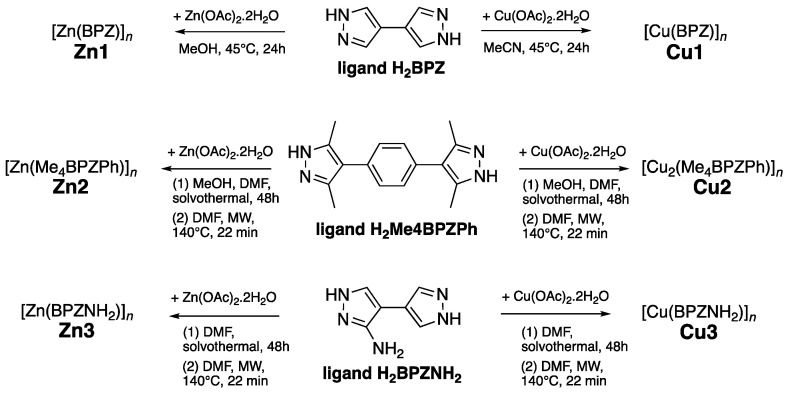
Synthetic procedure for **Zn1-Zn3** and **Cu1-Cu3** MOFs. For **Zn2-3** and **Cu2-3,** two different procedures were employed, indicated as (1) solvothermal synthesis and (2) the new procedure based on the use of MW.

Synthetic procedures for **Zn1-Zn3** and **Cu1-Cu3** MOFs are depicted in Scheme 1. Briefly, **Zn1** and **Cu1** were synthesized according to previously published methods, simply by stirring at room temperature a solution containing the bis(pyrazole)-based proligand in the presence of a base, to which the metal salt is subsequently added [26]. **Zn2**-**Zn3** and **Cu2**-**Cu3** were previously synthesized using the solvothermal condition in a high-pressure glass tube at 393 K for 48 h [27,28,29]. **Zn2**-**Zn3** and **Cu2**-**Cu3** have been obtained also by microwave-assisted synthesis (Scheme 1), with the aim of reducing reaction times, obtaining more crystalline products and higher yields. Indeed, as hoped, using the MAS conditions, the yields of **Cu2-Cu3** and **Zn2-Zn3** were significantly improved, resulting in total yields of 82% (**Cu2**), 91% (**Cu3**), 79% (**Zn2**) and 94% (**Zn3**), compared to previously reported efficiencies of 50%, 78%, 64% and 86%, respectively.

**Zn1** and **Cu1** were previously structurally characterized and found to adopt a 3D porous network containing 1D channels of square and rhombic shape, respectively. The Zn(II) ions are coordinated, within tetrahedral stereochemistry, by four N atoms of four exo-tetradentate (BPZ)^2−^ ligands, while in the case of Cu(II), the (BPZ)^2−^ spacers are coupled to give square planar stereochemistry on the metal node [26]. **Zn3** was characterized by a 3D porous network with tetrahedral metallic nodes and bridging (BPZNH_2_)^2−^ linkers representing the vertices and edges of square channels for Zn(II). **Cu3** showed square-planar metallic nodes and bridging (BPZNH_2_)^2−^ anions at the vertices and edges of the rhombic channels of a 3D porous framework [29]. **Zn2** probably has a crystal structure with tetrahedral ZnN_4_ nodes, to give chains connected to four neighbouring ones within a 3D porous framework, similarly to the isoreticular Co(II) MOF with the same (Me_4_BPZPh)^2−^ ligand [27].

Prior to their use in microbiological tests, all the samples were carefully activated through repeated washings with dichloromethane and prolonged heating at 353 K, to eliminate all traces of solvents incorporated in the pores of the MOFs. 

### 2.1. Antibacterial Activity

The activity of **Zn1-Zn3** and **Cu1-Cu3** against *E. coli* and *S. aureus*, chosen as models of Gram− and Gram+ bacterial strains, was assessed by enumerating the total colony-forming units (CFUs) after incubation for 24 h at 335 K. A preliminary investigation was carried out on our MOFs to determine the minimum inhibitory concentration (MIC) values for each of them, against the selected bacterial strains. In this context, different concentrations have been chosen: 5, 10, 30, 50, 100, 500 and 1000 μg/mL. In general, the MIC values for all compounds have been observed to be in the range of 10–30 μg/mL for both bacteria (Table 1), thus revealing a bacterial inhibition comparable to that of commonly used chemical disinfectants [30]. Furthermore, the values obtained are lower than those of the corresponding copper and zinc ions released by the corresponding metal salts (nitrates) [31]. The antibacterial MIC test conducted on **Zn2-Zn3** and **Cu2-Cu3** obtained in solvothermal conditions showed comparable results with respect to those with MOFs obtained with the MAS method. 

**Figure 1:**
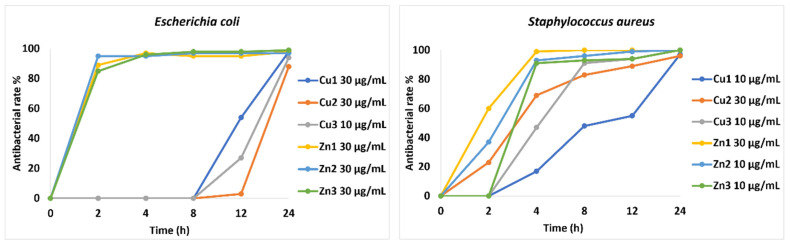
Antibacterial rate of **Zn/Cu-MOFs** as a function of time against *E. coli* and *S. aureus*.

The bactericidal and bacteriostatic effects against *E. coli* and *S. aureus* were verified by evaluating the temporal evolution of the antibacterial activity of the MOFs in the MICs, through the growth inhibition assay. Bactericidal activity is defined as the reduction in bacterial growth ≥ 99.9% within 18–24 h of inoculation, whereas bacteriostatic activity is associated with the reduction in bacterial growth maintaining stable between 90 and 99% within 18–24 h from inoculation [32]. Figure 1 shows the trend of growth inhibition of MOFs against the two bacterial strains, represented as a percentage of antibacterial rate over time. In general, the results show a good activity for all compounds at the defined concentrations against both strains. In detail, all the compounds are bacteriostatic after 24 h against *E. coli*, except for **Cu2**. On the other hand, **Cu1** and **Cu2** are bacteriostatic within 24 h against *S. aureus*, while the remaining compounds show not only a bacteriostatic action, but also a bactericidal one within 24 h of the experiment. 

Normally, to be effective, a biocide must bind to the bacterial cell wall, which is different in Gram+ and Gram− bacteria. Gram− bacteria (*E. coli*) have a membrane barrier outside the cell wall, formed by lipopolysaccharides (LPS) and proteins, while Gram+ bacteria (*S. aureus*) have a less complex cellular structure [33]. Based on this, the slightly lower performance observed against *E. coli* compared to *S. aureus* could be explained by considering the cell structure of the bacteria. 

### 2.2. Antibacterial Mechanisms and Morphological Study

**Figure 2:**
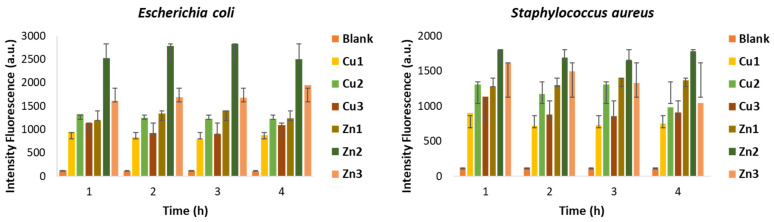
Formation of ROS in *E. coli* and *S. aureus* exposed to **Zn/Cu-MOFs** for 4 h (the data are represented as the mean ± SD of at least three separate experiments).

To further investigate the antimicrobial effect, studies focused on understanding the mechanism of action of **Zn/Cu-MOFs** against selected bacteria were conducted. In detail, the assay for the detection of reactive oxygen species and the analysis of the membrane permeabilization of the treated bacterial cells were performed. Moreover, to analyse morphological changes in both bacterial strains after the treatment with our **Zn/Cu-MOFs**, SEM was employed. One of the antibacterial mechanisms could be related to the oxidative stress of cells in contact with MOFs. Thus, the resulting cell damage is quantified by measuring ROS generation through the DCFH-DA probe. Initial diacylation of DCFH-DA by cellular esterase, followed by oxidation by ROS, leads to the formation of DCF, which is highly fluorescent and is detected by excitation/emission fluorescence spectroscopy at 485 nm/535 nm. Figure 2 shows the quantification of ROS generated after incubation of the bacterial strains with **Zn1-Zn3** and **Cu1-Cu3** for different time intervals (1–4 h). The amount of ROS was estimated by measuring the fluorescence emission at 528 nm. The results confirm the generation of free radicals in both bacterial strains, while the fluorescence emission of the untreated bacterial suspension (blank) is negligible. In general, all zinc MOFs show greater activity than copper MOFs against both bacterial strains, confirming the growth inhibition data. Particularly, a very rapid antibacterial activity of **Zn2** was confirmed for both bacteria by the fluorescence intensity measured after only one hour of treatment.

**Figure 3:**
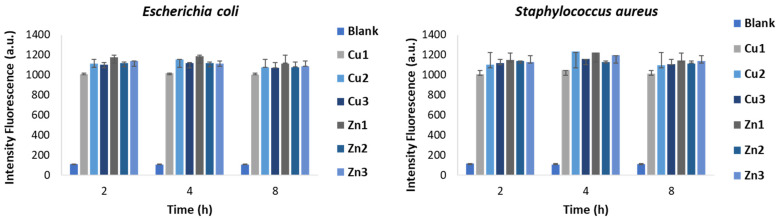
PI fluorescent emission for *E. coli* and *S. aureus* exposed to **Zn/Cu-MOFs** for 8 h (the data are represented as the mean ± SD of at least three separate experiments).

To evaluate the level of damage related to membrane permeability, the fluorescent PI staining assay was carried out. PI is an appropriate probe to assess membrane integrity, as it penetrates only the cells with damaged membrane. Once inside the cell, PI binds to DNA by intercalating between the bases with no preference. After binding, the fluorescence is enhanced and excited at 488 nm, resulting in emission maximum at 635 nm. The red fluorescence emission of bacterial cells exposed to **Zn1-Zn3** and **Cu1-Cu3** and stained with PI was measured against untreated bacterial suspensions placed in contact with PI. As shown in Figure 3, all MOFs tested at the MIC show, after 2 h of treatment, statistically significant damage in comparison with the control (blank) against both bacterial cultures. The fluorescence intensity remains higher than the blank even after 8 h of treatment against both bacteria. The results obtained verify the previous data, highlighting the ability of our MOFs to modify the permeability of the cell membrane, disorganizing it and leading to cell death. To further determine the overall viability of the bacterial cells, CLSM was performed by double fluorescence staining with Syto 9 and PI dyes. Based on the principle of staining, Syto 9 stains live and dead bacteria, which results in the production of green fluorescence. Similarly, dead bacteria emit red fluorescence upon staining with PI.

**Figure 4:**
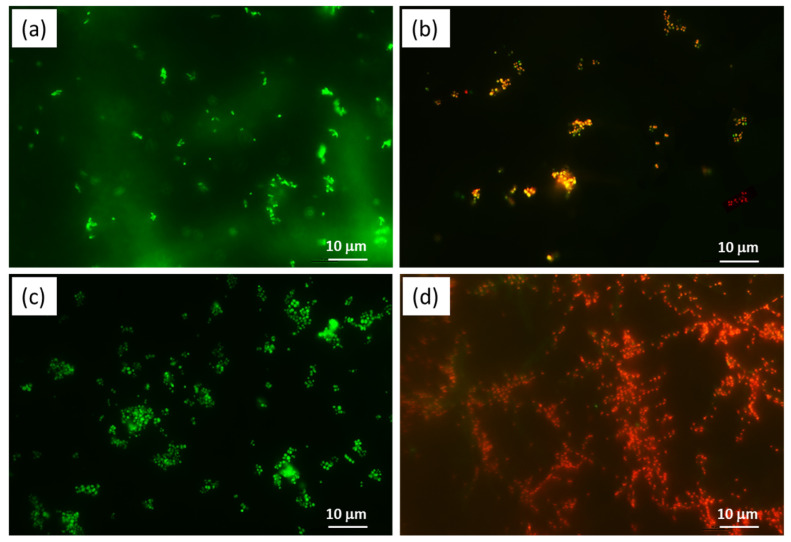
CLSM images of (**a**,**c**): *E. coli* and *S. aureus* control, respectively; (**b**) *E. coli* treated with **Cu1**; (**d**) *S. aureus* treated with **Zn1**.

The CLSM images in Figure 4 revealed that the untreated cells produced only sharp green light (Figure 4a,c), representing that the cells were alive and intact. On the other hand, the portion of cells tagged with red fluorescence was observed with **Zn1** and **Cu1** at the MIC (Figure 4b,d). This could be due to the increased permeability of the cell membrane after treatment. These results revealed that most of the cell membranes of *S. aureus* and *E. coli* were severely destroyed following **Zn1** and **Cu1** treatment at the MIC, which was in agreement with previous results. 

**Figure 5:**
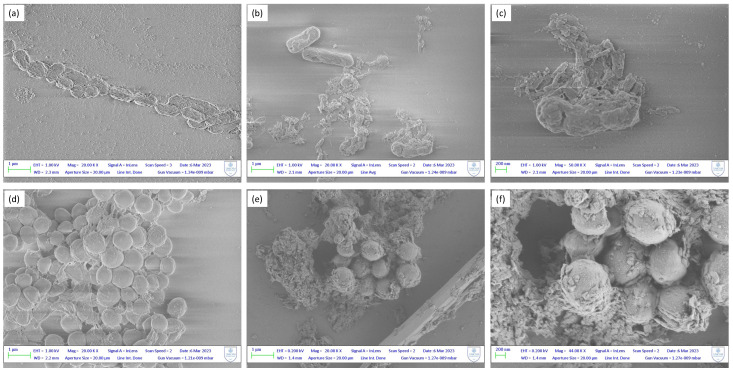
SEM images of (**a**,**d**): *E. coli* and *S. aureus* control, respectively; (**b**,**e**): *E. coli* and *S. aureus* treated with **Zn1** respectively; (**c**,**f**): magnification of the contact between **Zn1** particles and bacterial cell walls.

The structural alterations following the treatment of bacterial cells with MOFs was confirmed by SEM images. Figure 5 demonstrates the destructive impact towards the treatment of *E. coli* and *S. aureus* with **Zn1** compared to the untreated cells. The contact between the bacterial membrane and the unsaturated metal nodes of the MOFs’ surface is favoured by the presence of nanometric MOF particles, ranging from 50 to 100 nm. These are attracted to the negatively charged membrane, affecting its potential resulting in membrane disorganization and cell death. In Figure 5a, a control sample of *E. coli* exhibit regular rod-shaped morphology with a smooth and regular surface, and the cells are uniform in size and distribution. In contrast, MOF-treated cells showed an irregular and wrinkled outer surface, with a notable change also in cell size likely due to the agglomeration of MOF nanoparticles. In Figure 5d, a control sample of *S. aureus* shows a rigid and smooth cell wall with a round appearance and a regular size (so called “bald cell”). The clear presence of the biofilm matrix is interesting, which is typically formed by the *S. aureus* to ensure its survival. In Figure 5e,f instead, *S. aureus* shows a loss of the regular shape and dimensions after contact with the MOF particles, which again have the tendency to agglomerate around the bacterial membrane. Even with SEM images, it is therefore possible to confirm the previous results, thanks to the clear presence of the damage that occurs on the cell wall of the bacteria.

ICP-OES was used to determine the copper and zinc ions’ release ability for the investigated MOFs. It was found that the concentrations of copper(II) and zinc(II) ions, released from **Cu1** and **Zn1**, respectively, were 0.008 and 0.010 ppm. From the values obtained, it is possible to exclude the possibility that cellular damage may derive from the release of copper and zinc ions, because these are clearly lower than the MIC values found in the literature for both investigated ions. We can therefore hypothesize that the antibacterial mechanism is mainly due to the electrostatic interactions with the positive charges of the unsaturated metal nodes present on the surface of the MOFs, also known as the “chelation theory”. These interactions lead to cell membrane disorganization resulting in cell disruption. Furthermore, **Zn1-Zn3** and **Cu1-Cu3** seem to be able to induce the production of ROS leading to the breakdown of cellular integrity.

## 3. Materials and Methods

All chemical reactants were obtained from commercial suppliers (Merck) and employed without further purification. All solvents were purified by conventional procedures and stored under nitrogen prior to their use. All reactions and manipulations for the syntheses of bis(pyrazole)-based proligands H_2_BPZ, H_2_BPZ-NH_2_ and H_2_Me_4_BPZPh, and their interactions with zinc and copper acetate dihydrate, were carried out in the air, under ambient, solvothermal or microwave conditions. The solvothermal syntheses were carried out with flexiWAVE (Advanced Flexible Microwave Synthesis Platform) of the “MILESTONE” (Brondby, Denmark). The preparation of samples for microanalyses required drying in vacuo to constant weight (293 K, ca. 0.1 Torr). Elemental analyses (% m/m C, H, N) were performed with a Fisons Instruments 1108 CHNS-O Elemental Analyser (Glasgow, UK). Melting points are uncorrected and were recorded on a STMP3 Stuart scientific instrument and on a capillary apparatus. Infrared spectra were recorded from 4000 to 200 cm^−1^ with a PerkinElmer Spectrum 100 FT-IR instrument (Waltham, MA, USA). ^1^H, NMR spectra of ligands were recorded with a 500 Bruker Ascend^TM^ (500 MHz for ^1^H) instrument operating at room temperature relative to TMS. Thermogravimetric analyses (TGA) were carried out in a N_2_ stream with a PerkinElmer STA 6000 simultaneous thermal analyser. The samples were heated from 303 to 923 K at a heating rate of 10 K min^−1^ under a nitrogen flow (20 mL min^−1^). Viability assays on bacterial and fungal cultures were performed with a confocal microscope Nikon ECLIPSE Ti (Tokyo, Japan). The Optical Density of the bacterial suspensions (OD600) was determined by using a BioTek mQuant MQX200 Microplate Spectrophotometer (Winooski, VT, USA) at 600 nm.

### 3.1. Synthetic Procedures

#### 3.1.1. Synthesis of Proligands

The proligands 4,4′-bipyrazole (H_2_BPZ), 1,4-bis-4′-(3′,5′-dimethyl)-pyrazolylbenzene (H_2_Me_4_BPZPh) and 3-amino-4,4′-bipyrazole (H_2_BPZNH_2_) were synthesized by following the methods reported in the literature [29,34,35]. 

#### 3.1.2. General Procedure for Synthesis of MOFs Zn1-Zn3 and Cu1-Cu3

MOFs **Zn2-Zn3** and **Cu2-Cu3** were initially synthesized according to previously published procedures through a solvothermal synthesis (in a Teflon-lined stainless steel autoclave under autogenous pressure) in DMF at 393 K for 48 h [27,29]. **Zn1** and **Cu1** were prepared by mixing the metal acetate and H_2_BPZ in methanol or acetonitrile, respectively, in the presence of sodium methoxide and, after 1/2 h of gentle warming at 318 K, the solution was left under stirring at room temperature for 24 h. The precipitate obtained was filtered off, washed twice with methanol or acetonitrile, and, after repeated washings with dichloromethane, it was outgassed in high vacuum (0.005 mbar) at 353 K overnight [26]. **Zn2-Zn3** and **Cu2-Cu3** were also prepared following a MAS procedure. In detail, zinc or copper acetate (0.4 mmol) was added to a solution of 1,4-bis(3,5-dimethyl-1H-pyrazol-4-yl)benzene (0.106 g, 0,4 mmol) or 3-amino-4,4′-bipyrazole (0.060 g, 0,4 mmol) in DMF (20 mL). The resulting clear solution was poured into a 100 mL Teflon vessel. The reaction mixture was heated at 413 K, 800 W, under stirring for 20 min, then it was cooled down to room temperature in 14 h at 600 W. The resulting precipitate was filtered off, washed with hot DMF, solvent-exchanged with CH_2_Cl_2_ and air-dried. The yield was significantly improved, resulting in total yields of 82% (**Cu2**), 91% (**Cu3**), 79% (**Zn2**) and 94% (**Zn3**), with respect to previously reported yields of 50%, 78%, 64% and 86%, respectively. It is interesting to note that despite a different synthesis methodology, the BET values still remain high for both MOFs. 

### 3.2. Antibacterial Studies

#### 3.2.1. Bacterial Culture

The antibacterial activity of **Zn1-Zn3** and **Cu1-Cu3** was investigated against Gram-positive bacteria Staphylococcus aureus (*S. aureus*) ATCC 25923 (PBI International) and Gram-negative Escherichia coli (*E. coli*) ATCC 25922 (PBI International). Bacterial inocula were prepared with monocolonies of *E. coli* or *S. aureus* grown aerobically at 335 K overnight using tryptone soya broth (TSB) as the growth medium. For sterilization of the tubes, an Alfa-10-plus autoclave (PBI International) was used, operating at 394 K for 15 min. Bacteria proliferation was monitored by measuring the increase in optical density in the culture determined by using a BioTek mQuant MQX200 Microplate Spectrophotometer suspension at 600 nm (OD_600_). The enriched culture (log phase) obtained was further diluted to 10^6^ CFUs/mL concentration.

#### 3.2.2. Minimal Inhibitory Concentration (MIC)

The Minimum Inhibitory Concentration (MIC) is defined as the lowest concentration at which a compound is capable to inhibit the proliferation of the bacteria. The MIC determination of **Zn1-Zn3** and **Cu1-Cu3** was performed by using the broth dilution method with minor modifications [36] against Gram-positive bacteria Staphylococcus aureus and Gram-negative Escherichia coli. Briefly, each compound (10 mg) was dissolved in physiological solution (10 mL), then sonicated, to obtain a dispersed homogeneous stock solution. Serial dilutions were performed to test different concentrations (5, 10, 30, 50, 100, 500, 1000 μg/mL) for the determination of MIC values. An equal concentration of each bacterial inoculum (10^6^ CFUs/mL), previously obtained by a direct colony suspension in tryptone soya broth (TSB) of an overnight culture, was added to each sample. After incubation for 18–24 h at 335 K, the final solution was included uniformly into Petri dishes containing Plate Count Agar (OXOID). 

#### 3.2.3. Growth Inhibition Assay

To evaluate the antibacterial rate over time, the colony-forming units were measured by using the Agar diffusion method (Bauer et al., 1966). The test was performed using the MICs of each compound, which were dispersed in autoclaved physiological solution and to which 1 μL of the enriched culture (10^6^ CFUs/mL) was then added. All the Eppendorf tubes were kept on an IKA KS 130 BASIC platform shaker for 24 h at a speed of 160 rpm. To obtain the bacterial colony count, the final solutions were diluted and included into Petri dishes containing Plate Count Agar (PCA). Maintaining the same procedure, an untreated sample strain was used as a control (blank). The tests were carried out at different time intervals (0, 2, 4, 8, 12, 24 h) for mortality measurements over time. The growth inhibition was reported as the antibacterial rate (%) using Equation (1):

((CFUt_0_ − CFUt_k_)/(CFUt_0_)) × 100 (1)

where t_0_ is the zero time at the beginning of the experiment and t_k_ is the specific time in hours of the rate withdrawal during the experiment.

### 3.3. Antibacterial Mechanism and Morphology Study

#### 3.3.1. Reactive Oxygen Species (ROS) Detection Assay

The production of ROS was evaluated using a fluorescent-dependent method where the oxidant sensitive probe, 2′,7′-dichloro-dihydro-fluorescein diacetate (DCFH-DA), enhanced fluorescence under the condition of oxidative stress generation. Briefly, cells grown aerobically in TSB overnight and diluted to a concentration of 10^6^ CFUs/mL were incubated for 30 min with DCFH-DA at 335K. An amount of 10 μL of bacterial suspension was then added to the Eppendorf containing 10 μg or 30 μg of **Cu1-Cu3** and **Zn1-Zn3**, suspended in 1 mL of physiological solution. At the end of the treatment time, 100 μL of the suspension from each Eppendorf tube was transferred into 96-well plates, in triplicate, in the dark. The treatment was performed to evaluate the production of ROS after 1, 2, 3, 4 h. Maintaining the same procedure, an untreated sample strain was used as a control (blank). Fluorescence emitted by each well was read using the FLUOstar Omega fluorescence cytometer from BMG LABTECH at 485/20 nm and 528/20 nm wavelengths for excitation and emission, respectively. The average of the triplicate was calculated and reported as the intensity of fluorescence in arbitrary units (a.u). 

#### 3.3.2. Propidium Iodide Uptake

Bacterial viability staining treated with compounds **Cu1-Cu3** and **Zn1-Zn3** was assessed using Propidium Iodide (PI) as a red fluorescent dye, which penetrates only damaged cellular membranes. A suspension (10^6^ CFUs/mL) of *E. coli* and *S. aureus*, treated with 10 μg or 30 μg of **Cu1-Cu3** and **Zn1-Zn3**, was incubated for a period of 24 h at 335 K. After 4, 8 and 12 h, 100 μL of the content was poured in triplicate into a 96-well plate in the dark and 1.5 μL of Propidium Iodide (PI) was added in each sample well. Adopting the same protocol, an untreated sample strain was used as a control (blank). Fluorescence emitted was read using the FLUOstar Omega fluorescence cytometer at 485/20 nm and 528/20 nm wavelengths for excitation and emission, respectively. The average of the triplicate was calculated and reported as the intensity of fluorescence in arbitrary units (a.u). 

#### 3.3.3. Confocal Laser Scanning Microscopy (CLSM) Study

To evaluate the level of damage related to cell membrane permeability caused by **Zn1** and **Cu1** (arbitrary chosen), CLSM analysis was performed with LIVE/DEAD^®^ BacLight™ Bacterial Viability Kits (Invitrogen, Waltham, MA, USA). BacLight is composed of two nucleic acid-binding stains: SYTO 9™, which penetrates all bacterial membranes and stains the cells green, and PI, which penetrates cells with damaged membranes, and stains the cells red [37]. A bacterial suspension in the range of 1 × 10^8^ CFUs/mL (0.3 OD_670_) for *E. coli* and 1 × 10^7^ CFUs/mL (0.15 OD_670_) for *S. aureus* was treated with 10 μg of **Zn1** and **Cu1**, during the logarithmic growth phase, for 4 h. Then suspensions were centrifugated at 10.000 rpm for 15 min. The supernatant was removed, and the pellet was resuspended in 0.85% NaCl solution. After the procedure was repeated three times, a mixture of Syto 9 and PI was prepared and added to all samples in the dark. They were incubated for 15 min in the absence of light and finally 10 μL of each sample was fixed between a slide and an 18 mm square coverslip to observe the fluorescence under confocal microscope (Nikon ECLIPSE Ti). The control assay was conducted without treatment. The excitation/emission for these dyes are 480/500 nm for SYTO 9 and 490/635 nm for PI.

#### 3.3.4. Scanning Electron Microscopy (SEM) Study

SEM was performed to study the physical and morphological variations in bacterial cells after their treatment with **Zn1** and **Cu1** MOFs, arbitrarily chosen. Bacterial strains were processed according to the following procedure: briefly, log phase cells of *E. coli* and *S. aureus* (10^6^ CFUs/mL) were incubated with 1 mg of **Zn1** and **Cu1**, for 4 h at 335 K. After the incubation, bacterial cells were centrifuged, washed thrice with fresh physiological solution, followed by glutaraldehyde (2.5%, *v*/*v*) fixation for 2 h on silicon plates. The latter were dehydrated in ethanol (10%, 30%, 50%, 70%, 90%, 100%, *v*/*v*). Finally, the silicon plates were placed on aluminium stubs using a self-adhesive carbon conductive tab, and they were chrome-coated. The bacterial cells were studied on a SEM operating at 1–5 kV. The unprocessed bacterial cells were chosen as control samples.

#### 3.3.5. Copper and Zinc Ion Release Test

The concentrations of released copper and zinc ions were measured under conditions amenable to biological testing, by Inductively Coupled Plasma Optical Emission Spectrometry (ICP-OES). The samples **Zn1** and **Cu1** were arbitrarily selected and 1 mg of each was suspended in 10 mL of physiological solution for 72 h at 335 K. Finally, the suspensions were filtered through a pad of Celite due to the small grain size of the samples, and the maximum quantity of released ions was evaluated.

## 4. Conclusions

The present work reports the results of the investigation of antibacterial activity of six Zn(II) and Cu(II) MOFs with three different bipyrazolate linkers. We carried out for the first time a detailed study on the antibacterial potential of these families of MOFs and demonstrated that it is possible to use them as antibacterial agents due to their significant activity against *E. coli* and *S. aureus*, chosen as models of Gram− and Gram+ bacterial strains. Particularly, Zn-MOFs achieve within the first hours of exposure a great viable cell reduction in both strains. The antibacterial mechanism of **Cu1-Cu3** and **Zn1-Zn3** was suggested to be the disruption of the integrity of the bacterial cell membrane and generation of ROS through stress induced by contact with the MOFs’ surface. Among them, **Zn1** was found to be the best candidate for future applications. For this reason, we deeply investigated its possible antibacterial mechanism. The “contact” hypothesis with damage of the bacterial membrane was also confirmed by the significant morphological alterations observed in the SEM images, where a change in the regular shape of the bacteria is clearly visible, due to the agglomeration of the MOF particles around the cell membrane and to the established electrostatic interactions. Damage to the cell membrane permeability occurs without the release of metal ions. Finally, the total yield for **Cu2-Cu3** and **Zn2-Zn3** MOFs was improved by exploiting the microwave-assisted synthesis, significantly decreasing their reaction times. 

## Figures and Tables

**Table 1:** Antibacterial activities of **Zn/Cu-MOFs** evaluated by MIC (μg/mL) compared with those of copper and zinc nitrates.

Compound	*E. coli*	*S. aureus*
Zn1	30	30
Zn2	30	10
Zn3	30	10
Cu1	30	10
Cu2	30	30
Cu3	10	10
Zn^2+^	50–100	50–100
Cu^+/2+^	50–100	50–100

## Data Availability

No supporting data are present.
